# Use of a Modelling Tool to Optimise the Organisation of the Demand for Primary Oral Health Care in the Brazilian Unified Health System

**DOI:** 10.3290/j.ohpd.b4836027

**Published:** 2024-01-15

**Authors:** Katia Miyuki Sasaki, Talitha Giovanna da Silva Neres, Erica Tatiane da Silva, Jorge Luis Lopes Zeredo

**Affiliations:** a Health Management Analyst, Evidence Program in Policies and Health Technologies, Oswaldo Cruz Foundation, FIOCRUZ Brasilia, Brasília, Brazil. Idea, experimental design, analysed the results, wrote the manuscript, proofread the manuscript.; b Resident, Multiprofessional Residency Program, Oswaldo Cruz Foundation – FIOCRUZ Brasilia, Brasília, Brazil. Analysed the results, proofread the manuscript.; c Public Health Researcher, Evidence Program in Policies and Health Technologies, Oswaldo Cruz Foundation, FIOCRUZ Brasilia, Brasília, Brazil. Experimental design, advisor, proofread the manuscript.; d Associate Professor, Graduate Program in Health Science and Technology, University of Brasilia, Brasilia, DF, Brazil. Advisor, proofread the manuscript.

**Keywords:** demand, primary health care, oral health, workflow

## Abstract

**Purpose:**

To describe the use of work process modelling to optimise the organisation of the demand for oral health treatment in primary care units in Brazil.

**Materials and Methods:**

The oral health care routine was at first described as the “AS IS” model, which was evaluated by the oral team professionals, rearranged, and further described as the “TO BE” model described using a business process management modelling tool. The significant increase in the demand of patients due to restrictions offered by the dental service in addition to non-urgent treatments being avoided by patients during COVID-19 pandemic was also considered.

**Results:**

Structuring the work processes in a visual way using modelling tools was useful to picture the entire treatment process and adjust when needed. The use of the managerial tool was useful to understand and reorganise the workflow of organising the demand and ultimately improve the efficiency of the resources. The use of such managerial tools helped oral health professionals to efficiently rearrange their tasks and set priorities to meet their needs.

**Conclusions:**

With the use of management tools, each unit can readjust its structures and ways of working, aiming to improve the quality of public health care services provided to patients.

In 2021, the 74th World Health Assembly of the World Health Organization (WHO) placed oral health on the global health agenda. The resolution urges countries to consider a variety of actions, with implementation dependent on each country’s particular set of circumstances. Some of the key points included integration of oral health into national health policy, a focus on preventive strategies as opposed to approaches that emphasise treatment of existing disease, and consideration of workforce models that maximise efficiency.^12^

In Brazil, oral health has its own policy, the National Oral Health Policy, also known as the Smiling Brazil Programme, launched in 2003. This policy was conceived to ensure the public’s access to oral health care and overcome the inequalities brought about by the traditionally hegemonic, curative and illness-centred care. Hence, the policy is composed of a series of actions to promote, prevent, and recover the oral health of the citizens.^11^ These actions aim to rearrange primary oral health care in order to provide oral health care through the Brazilian Unified Health System to the population at large, particularly by the inclusion of an oral health team in the family health strategy.^15,16^ The Smiling Brazil Programme is a multi-pronged approach and recognises that overcoming oral diseases requires a combination of patient education, as well as disease prevention and treatment. By targeting various aspects of oral health care and engaging communities, the programme strives to reduce the burden of oral diseases and improve the oral health and well-being of the Brazilian population.^13,17,21^

In the Federal District (Brasilia), the local government’s Department of Health has aimed to restructure standards and guidelines for oral health primary care as indicated in the family health strategy model. Oral health teams were deployed, and the levels of oral health care were adjusted based on optimised management and efficiency of the health care networks.^3^ The guideline states that it is the teams’ responsibility to minimally perform the clinical examination, the vulnerability assessment, and the necessary outpatient procedures to effectively meet the demand, according to the evaluation of the oral surgeon. However, organising the demand is recognised as one of the leading problems faced in the daily routine of oral health services; therefore, the strategies used for such classification should be discussed among users and healthcare workers. The criteria for case prioritisation should not be that of order of arrival, but the severity of the case and the level of pain.^14^ The objective of vulnerability assessment is to allow the prioritisation of care to those with higher risk or higher disease activity during treatment.^4^ Thus, organising the demand for oral healthcare is a major challenge for the oral health teams, because it needs careful planning and requires time and communication with other professionals. 

Accordingly, it is necessary to understand the entire process to start planning and improving actions to respond to the needs and changes that always occur in the workplaces. Lately, using managerial techniques, such as the Business Process Management (BPM), has been suggested as a feasible and useful methodology to design and optimise clinical processes in the health field.^6^ BPM is defined as a subject that combines business perspectives with the goal of improving the organisation’s operation. It increases the effectiveness and efficiency of an organisation and contributes significantly to overall performance and competitiveness by standardising organisation processes.^23^ The business process modelling tools are used to analyse the status quo (“IS” status) and simulate how they should be carried out to achieve better results (“TO BE”). These tools are useful to identify the steps of the processes in addition to the people responsible for each one of them.^18^

The present study aims to describe the organisation of the demand for oral health primary care before and after using managerial tools to help restructure and improve the routines of a primary oral health team in the Federal District (Brasilia) in Brazil.

## MATERIALS AND METHODS 

This is a descriptive, qualitative study carried out in accordance with the consolidated criteria for reporting qualitative research (COREQ), when applicable.^22^ It included primary care units (PCU) of the Federal District in Brazil. The theoretical frameworks underpinning the present study were documents such as official regulations, manuals, and guidelines, as well as the documents and processes themselves that were generated by the participants throughout the research. The instrument used to collect data was a semi-structured in-depth interview with open-ended questions followed by more in-depth interviews when necessary. The interviewer (a female health professional, specialist in public management) was trained by the main researcher (a female dental surgeon with a PhD in dentistry, a specialist in public management and health organisations management). Before the interview, the interviewer introduced herself and presented the goals of the study together with the informed consent form to be signed if consent was given.

Out of the thirteen units that met the inclusion criteria, three were excluded: two for being in rural areas and one for not responding. Additionally, two professionals declined to participate, and because they were the only dentists in charge of their respective units, those two units were also excluded. In total, we mapped eight units. All participants were selected by convenience criteria and the method of exhaustion was used to include all eligible subjects within the determined population. Recruitment was restricted by the saturation criterion, particularly at units with more than one dentist. The inclusion criteria consisted of oral surgeons who were part of a primary care oral health team and who agreed to participate in the study. Data collection took place from October 2020 to July 2022. The subjects were contacted after the study was presented to the regional coordinator of public health services, and was initially made by phone to set an appointment with the surgeon who arranged the meeting. 

The workflow processes during demand organisation were mapped out according to the information from semi-structured, in-depth interviews with open-ended questions. Interviews were carried out between the examiner and interviewee only. An interview guide was applied that contained questions about access to oral health services, patient welcoming, vulnerability assessment, disease risk classification, care upon spontaneous demand, care outside the coverage area; continuity of care, scheduled demand, booking appointments and absenteeism. All interviews began with an explanation of the purpose of the interview – to gain an understanding of how demand was organised. The interviewer stressed that certain points of the organisation are subjective and that there are no correct or incorrect answers. Additionally, it was emphasised that the interview aimed to observe how each professional organises their demands based on their individual circumstances, including their creativity, in order to arrive at optimal solutions to problems.

The first question was “How is spontaneous and scheduled demand organised in your unit?”, so that the professional could explain the relevant routines. Subsequently, if certain aspects were not covered in the initial response, we asked tailored questions related to the routines. These included: When the patient arrives at the unit, who serves as the patient’s initial point of contact? Who gives instructions on how to proceed? After the first contact, who will attend to the patient next? Is the patient referred directly to the dental office? If yes, who is the designated professional responsible for greeting the patient upon arrival to the dental office? If not, where is the patient referred to? How is this reception process conducted in the dental department? Upon arrival, does the patient receive instructions from a technician or other professional? If so, what instructions are given? What criteria are utilised for these instructions? Does the technician use an instrument such as a questionnaire, chart or system record to identify/register the patient? What priorities do you attend to? What are the priority categories for care? Can dental patients arrive at any time and are they always admitted? What hours are allotted to receiving spontaneous demand? How is immediate dental care managed upon identification of a dental emergency? Are there any criteria utilised for handling spontaneous demand, such as the risk classification from the guideline for dental surgeons? Once the patient’s dental emergency has been resolved, how will a follow-up appointment be scheduled (pertaining to how scheduled demand is managed)? In terms of scheduled demand, how is it being met? If the patient comes to the unit for dental treatment, is there a specific day for this appointment? If so, when? If not, why? Are there criteria for prioritising dental care? If so, which are the priority groups? Are pregnant women, children aged 0-5, those with cardiovascular risk, diabetes, hypertension, smoking, or obesity prioritised? Is there a list of patients to be assisted that is being compiled? About scheduling patients for dental treatment, is scheduling done by appointment or by shift? If scheduling is by shift, what is the order of attendance? Further questions were asked as necessary in order to clarify points related to the organisation of demand. The questions “What do you think are the biggest problems and obstacles in carrying out these processes?” and “What suggestions do you have for improvement?” were made after each topic to build the “TO BE” model. The particularities of each team and the emphasis on the strategies that, in their opinion, are working properly were recorded.

A pilot interview was carried out before fieldwork with oral surgeons from units not included in the present study, to adjust and refine the questions. The interview had no time limit and the duration averaged 30 min. The interviews were recorded with permission of the participants and used solely to keep a record of answer details. 

**Fig 1:**
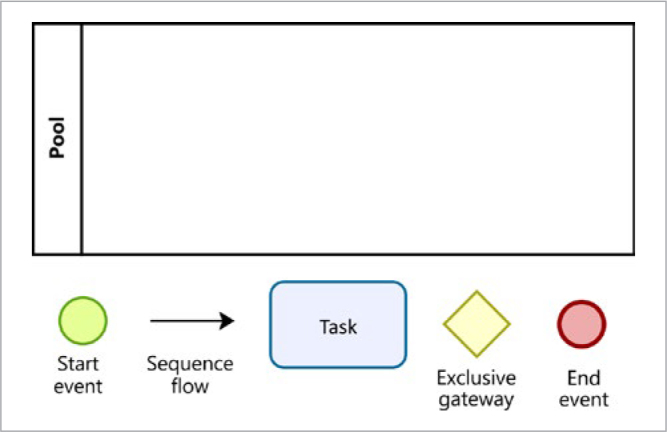
Symbology for the modelling: pool and basic elements of BizAgi Process Modeler. Green circles represent start of a process. Red circles represent the end of a process. Arrows represent the sequence flow. Rectangles with rounded corners represent the tasks. Diamond shapes represent the gateways.

The work processes were described and designed in the computer tool BizAgi Process Modeler version 3.6.0.044, 2019. The symbology used for this modelling is shown in Fig 1. The start and the end of a process are represented by the green and red circles, respectively. The arrows represent the sequence flow and the rectangles with rounded corners represent the tasks. Gateways are elements that create the division of the flow and represent the convergence or divergence of the continuity of a flow at the instant a decision is made. Gateways are represented as diamond shapes.

After the analysis of all eight workflows, we deliberately chose one unit due to its high level of detail and consistent flow based on the guidelines. All the professionals involved in the process were receptive to the changes and willing to improve their work. In this unit, the workflow at the first interview was described as the “AS IS” situation. 

After this first record, the whole process was analysed and extensively discussed among the oral-care team of professionals. The oral surgeon in charge, together with other health professionals involved, rearranged the flow to optimise the resources, redistributed tasks, and reduced waste and repetitions based on the resulting workflow obtained with the study. The oral-care team also took into consideration the fact that the unit faced a significant increase in patient demand due to restrictions from the dental service, in addition to non-urgent treatments being avoided by patients during the Covid-19 pandemic. This updated model is referred to as the “TO BE” situation.

The present study was performed in accordance with the guidelines and standards regulating research involving human beings approved by Resolution No. 466 of December 12, 2012, issued by the Brazilian National Health Council (CNS). This resolution consolidates bioethical benchmarks from the viewpoint of the individual and the communities, such as autonomy, non-maleficence, beneficence, justice, and equity, among others, and it seeks to ensure the rights and duties that refer to the participants of the research, to the scientific community and the State. Ethical approval was granted by the Research Ethics Committee of the Oswaldo Cruz Foundation Brasilia under the reference CAAE 10207719.7.3001.5553. Informed consent forms accompanied the proposal and data collection instruments to inform participants about the aims and to guarantee their privacy and confidentiality. Participants were presented with an overview of the project, as well as how the results were intended to be used academically at the local level. To ensure the confidentiality of their interviews, we removed all identifying information. All participants provided informed consent prior to taking part in the study.

## RESULTS

In the “AS IS” process, the organisation of the demand began with the security guard of the unit asking what kind of problem the user might have. If the demand was related to oral health, the security guard collected the user’s Brazilian Unified Health System card and inserted it in a small box next to the door of the oral health service’s office, otherwise the user was redirected toward the nurse’s office for triage. It was observed that the security professional workstation was strategically placed at the entrance of the unit, which made the user’s transit into the unit flow naturally towards the security professional. Moreover, the security workstation was adjacent to the oral health service’s office, which made this initial “triage” convenient, based solely on the complaint reported by the users. Notice that the “related to the mouth” criterium has always been used as a simple and objective way to separate oral health care demand from others. Throughout the daily routine care and between treatments, the oral health technician would check the outside box for the demand. Then, the technician organised the daily activities to collect the user’s card, consulted the Brazilian Unified Health System registry, and performed an initial interview to classify the user’s demand by priority. Users considered as “priority” were referred to the oral surgeon to evaluate whether they needed urgent care. Other users were referred to the oral surgeon to evaluate whether they needed urgent care after the priority users. If the evaluation by the oral surgeon indicated that immediate care was needed, the user started treatment on the same shift or no later than the next shift. An oral surgeon performed the evaluation and risk classification of the users each day, to determine the type of care that should be provided. Otherwise, the oral surgeon evaluated whether the complaint could be resolved on the same day and if there was an opening in the schedule. If a slot was available, the user was seen to resolve the main complaint (advanced or open access). If not, he/she was put on scheduled demand. After the resolution of the main complaint, if the user was from the area covered by the PCU, a follow-up appointment was made as needed. If the user did not belong to the PCU’s coverage area, the patient would be referred with the “Referral with Responsibility” (document with the patient’s data and description of the provided care) to his/her reference team. 

Pregnant women could have a different flow, as they were referred from the obstetric care team to the dental-care team to be followed-up throughout the gestational period. The oral follow-up was done on the same day as the prenatal appointments. The oral appointments were scheduled from the beginning of the prenatal period and marked as scheduled demand. 

**Fig 2:**
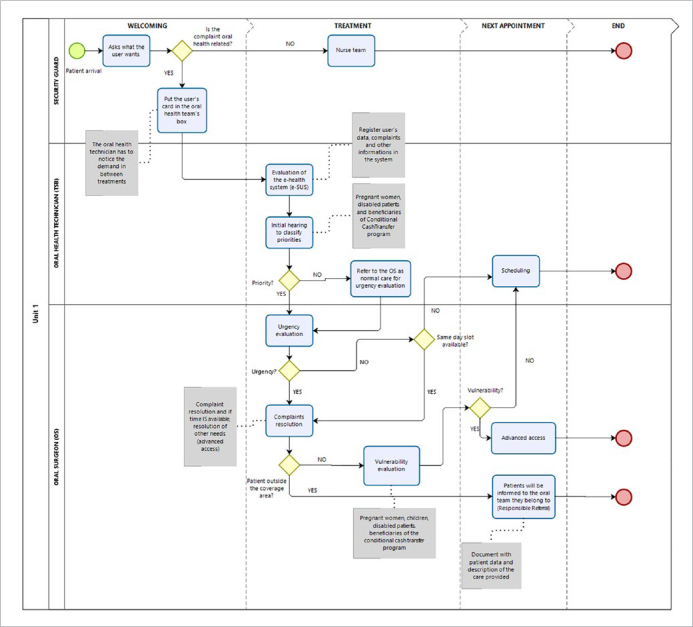
“AS IS” workflow of the demand organisation in Unit One. Work process related to the demand organisation in Unit One before the use of modelling for restructuring.

To meet the scheduled demand, the ratio between the demand for oral health care and the supply of vacancies was considered. In case the demand for oral care was higher than the vacancies available, oral health treatment focused on priority groups such as pregnant women, children from 0 to 5 years old, and patients with chronic diseases. When the ratio of supply and demand for care stabilised, the advanced access was resumed. This workflow was labelled “AS IS” and is shown in Fig 2.

**Fig 3:**
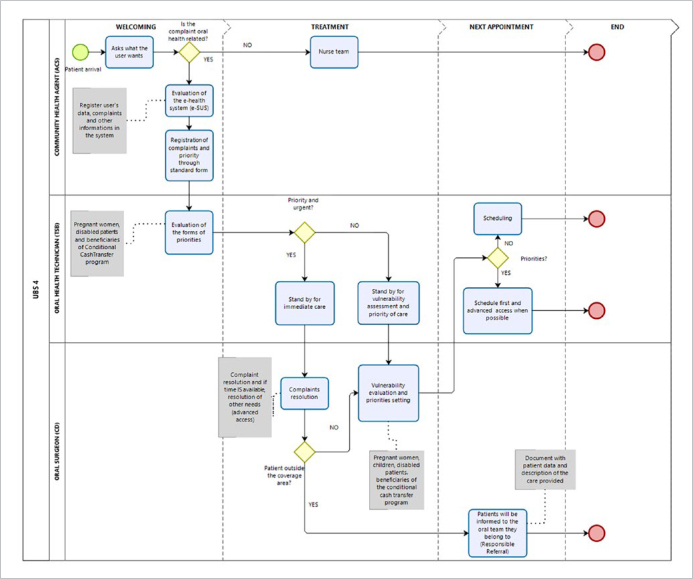
“TO BE” workflow of the demand organisation in Unit One. Work process related to the demand organisation in Unit One after rearrangement and reorganisation.

After remodelling, the process was called “TO BE” and is shown in Fig 3.

The first change was the inclusion of the community health agents in the process. The community health agents performed welcoming through a standard form. The form was elaborated and discussed together with all professionals involved to clarify how to fill it out. The questions are related to the main complaint and vulnerability and are straightforward and easy to understand. Having all the information written and compiled in groups helps in organising priorities. Furthermore, it is also in this step of the process that the patient is registered in the Brazilian Unified Health System. The oral health technician evaluates the priorities both regarding risk and vulnerability. The urgent-care and vulnerable patients are treated first. If the complaint is not urgent and the patient does not live in the covered area, they will be redirected to their own team with the “Responsible Referral”. However, in cases when urgent care is needed, the patient will receive treatment to meet the immediate demand. In this rearranged workflow, the previous registrations made by the community health agents saves the oral health technician time that can be spent helping the oral surgeon during treatments. The oral surgeon treats all the urgent cases of the day, and ideally would perform the advanced access, but since the Covid-19 pandemic, the ratio of demand to vacant treatment slots has always been too high to do so. Therefore, the ratio must be constantly revaluated to choose the best strategy to follow.

## DISCUSSION

The results presented the workflow of the organisation of oral health care demand considering user’s inequalities, e.g., based on lower socioeconomic backgrounds and other risk groups, as recommended in Brazil’s National Oral Health Policy and pointed out by the World Health Organisation.^2,12,19^

Although the “AS IS” process was well established and flowing, many shortcomings became clear after mapping. Therefore, the process of mapping generated data to redesign of workflows, optimise the work processes, and avoid losses and waste. In addition, the BPM helped to allocate the right professional to each task and enabled the development and improvement of the oral health team routine in organising the work demand. Accordingly, studies showed that the proper organisation of care and structured work processes can improve the performance of oral health services and promote better access to oral health in Brazil.^5^

In addition, the workflow presented in this study may prompt other oral health care units in the country to organise their demand in a similar fashion. It has been reported that organising the agenda to ensure a response to spontaneous demand and the continuity of care is necessary to increase access to oral health care.

The results obtained can support the responsible managers in making decisions related to improving the quality of oral care to the population and contribute to the quality management of oral health services offered. Likewise, previous studies showed that the use of business process management tools helped organise the demand of patients and consequently improved access to the health service.^18,20^

Recently, a study demonstrated that the provision of oral health care is associated with the organisation of the teams’ work, planning actions, and the characteristics of the oral surgeon,^7^ which corroborates the need for an analysis of the strategy used by each oral health team for improvements in work processes. Also, the Federal District’s Oral Health Guideline suggests that the way of organising the demand in each oral health care unit should be evaluated and, if necessary, adapted.^3^ In the present study, the initiative for changing and adapting the flow to meet the new, accumulated demand was from the oral surgeon, who showed interaction and alignment with teammates. 

Therefore, solely reorganising the levels of care and elaborating guidelines are not enough to offer a quality health service to the population, because organisational environments are complex and almost all processes undergo rapid and significant change. There is a need to continually review the organisation processes to respond quickly and appropriately to the needs of the population. Quality management and management by processes can be highlighted as managerial tools used to seek improvement in the quality of services, because they contribute to the increase in efficiency, efficacy, and effectiveness of public services.^6,9,10^

Accordingly, in the present study, structuring the work processes in a visual way using modelling tools was useful to picture the entire demand-organisation process and adjust when needed. The use of such managerial tools helped the oral health professionals to efficiently rearrange their tasks and set priorities to meet their needs. With the use of management tools, each unit can readjust its structures and ways of working, aiming to improve the quality of public health care services provided to patients. 

With process modelling, the visualisation of the whole process is facilitated and, in this case, showed that the technician in oral health was not being fully employed. In the suggested “TO BE” model, some of the activities were transferred to the community health agents, which provided more time for the technician to work with the dentist. Indeed, the productivity of the oral surgeon is known to be improved by both an oral health assistant and a technician’s assistance, which increases the efficiency of the service, expands coverage at lower costs, and prevents stress as well as professional fatigue.^1,8^

It is worth mentioning that this research was carried out during the COVID-19 pandemic, and according to the WHO reports, oral health services have been among the most affected essential health services, with 77% of the countries reporting partial or complete disruption. Hence, mapping the work processes may be a useful tool to help cope with the accumulated demand presented in the post-pandemic scenario. 

Our results are based on a limited number of interviewees and units. Thus, the information is rather subjective, and the conclusions might be influenced by the experience and observations of the researchers. To address these limitations, further investigation should be carried out in other units in Brazil and compared to other middle-income countries’ health systems. 

## CONCLUSION

Our findings contributed to the improvement in the work processes of the health units in the Federal District (Brasilia) and might be considered to help policy makers tackle the high demand for oral health care and inspire professionals during demand organisation in the Brazilian Unified Health System and beyond.
